# Bone Tissue Engineering and Regeneration

**DOI:** 10.1155/2014/137529

**Published:** 2014-07-22

**Authors:** Benjamin Levi, Bruno Péault, Aaron W. James

**Affiliations:** ^1^Department of Surgery, University of Michigan Health Systems, Taubman Center, 1500 East Medical Center Drive, XPC 5340, Ann Arbor, MI 48109-0219, USA; ^2^Orthopedic Hospital Research Center, David Geffen School of Medicine, University of California, Los Angeles, 615 Charles E. Young Drive South, Room 410, Los Angeles, CA 90095, USA; ^3^Center for Cardiovascular Science and MRC Center for Regenerative Medicine, University of Edinburgh, UK; ^4^Department of Pathology & Laboratory Medicine, David Geffen School of Medicine, University of California, Los Angeles, 10833 Le Conte Avenue, A3-251 CHS, Los Angeles, CA 90095, USA

Bone tissue engineering and regeneration constitutes a burgeoning new area of investigation, incorporating scientists, engineers, and clinicians with diverse academic backgrounds. With an aging global population, the need to engineer and regenerate mesenchymal tissues becomes even more pressing. This issue on bone tissue engineering and regeneration compiles 9 exciting manuscripts which reflect the diversity of this fascinating subject.

Bone tissue engineering can be conceived of as a harmonious intersection between different scientific fields of interest, including cell biology, cell signaling, and bioengineering/material sciences. Thus, effective therapies for bone engineering typically employ the coordinated manipulation of cells, biologically active signaling molecules, and biomimetic, biodegradable scaffolds. Bone tissue engineering has become increasingly dependent on the merging of innovations from each of these fields, as they continue to evolve independently. In the following special issue, we sought to incorporate these diverse areas of emphasis in order to reflect current trends in the field.

Three manuscripts dealt with cell based modalities of bone tissue engineering. J. Lisiecki et al. examine unique subpopulations of adipose-derived stromal cells (ASC). In their study, ASC were derived from the abdominal subcutaneous fat depot from either normal patients or those with a ventral hernia. Their results indicate important differences in the vasculogenic effect of ASC derived from different patient populations, emphasizing the need for scientists to consider patient comorbidities when they isolate various mesenchymal stem/stromal cell (MSC) populations. With a different focus, K. Lim et al. examined alveolar bone-derived MSC and the utility of low-intensity pulsed ultrasound for induction of MSC osteogenesis. Their results suggest that low-intensity ultrasound may actually improve the viability and osteogenic differentiation of alveolar bone-derived MSC, results that may well be applicable to other MSC populations. In another fascinating manuscript, M. H. Ng et al. used a novel combination of MSC with fibrin impregnated ceramics for leporine tibial bone regeneration. Here, they demonstrate that platelet-rich plasma in conjunction with bone marrow-derived MSC stimulates structural and functional recovery after segmental defect creation.

Three manuscripts dealt with advancing and optimizing the use of scaffolds for bone tissue engineering. C. J. D. Bergmann et al. described a rapidly manufactured bone substitute based on tricalcium phosphate, polyethylene glycol, and trisodium citrate that can be formed at room temperature. Their data, performed in a sheep model, suggest that this room temperature forming bone cement is osteoinductive/osteoconductive and promising for future clinical application. K. Nazemi et al. examined an amalgam of chitosan, bioactive glass and PLGA nanoparticles. Surface appearance, porosity, mechanical properties, and osteoinductivity were assessed. L. Chen et al. applied scaffold technologies to the engineering of cartilage, examining the fetal chondrocytes when placed on nanohydroxyapatite/PLGA composite scaffolds.

Two manuscripts focused on the development of model systems to test and optimize bone regeneration, an often-overlooked subject. L. Poser et al. described a middiaphyseal femoral defect in rats using a novel radiolucent plate for fixation. Examined in both osteoporotic and nonosteoporotic rodents, the radiolucent, biologically inert fixation device allows for standardized defect creation to ensure experimental reproducibility. In a similar study of critical sized bone defects, J. L. Lansdowne et al. described a bilateral nonhealing iliac wing defect in sheep. Such well-described and precise models facilitate comparisons between studies and between bone research groups.

By compiling these papers, we hope to enrich our readers and researchers in the field of bone tissue engineering and regeneration. We thank those authors that participated in the present special issue.

*Benjamin Levi* *Bruno Péault* *Aaron W. James*

